# A swallowing and breastfeeding intervention programme for small and sick neonates embedded in kangaroo mother care

**DOI:** 10.4102/sajcd.v71i1.1055

**Published:** 2024-08-09

**Authors:** Alta Kritzinger, Elise van Rooyen, Anne-Marie Bergh

**Affiliations:** 1 Department of Speech-Language Pathology and Audiology, Faculty of Humanities, University of Pretoria, Pretoria, South Africa; 2 Department of Paediatrics, Faculty of Health Sciences, University of Pretoria, Pretoria, South Africa; 3 Kalafong Provincial Tertiary Hospital, Pretoria, South Africa; 4 Research Centre for Maternal, Fetal, Newborn and Child Health Care Strategies, Faculty of Health Sciences, University of Pretoria, Pretoria, South Africa; 5 Maternal and Infant Health Care Strategies Unit, South African Medical Research Council, Faculty of Health Sciences, University of Pretoria, Pretoria, South Africa

**Keywords:** kangaroo mother care, neonatal care, oral feeding difficulties, oral sensorimotor intervention, practice guidelines, preterm and low-birthweight neonates, small and sick neonates, speech-language therapy, swallowing and breastfeeding intervention programme

## Abstract

Available evidence of oral sensorimotor interventions for small neonates is not strong. Evidence of interventions for sick term neonates is largely lacking. Studies are limited by risk of bias and inconsistency. Evidence of interventions relying on a single stimulation technique only appears to be low to very low. Ongoing research is required.

**Contribution:**

We describe a five-component neonatal swallowing and breastfeeding intervention programme embedded in the practice of kangaroo mother care (KMC). Drawing on oropharyngeal physiology, neonatology, neurodevelopmental care, breastfeeding- and KMC science, the programme is the product of collaboration between a speech-language therapist and a medical doctor, and their team. Its implementation is dependent on coaching mothers and the neonatal care team. Researchers are invited to determine outcomes of the programme.

## Introduction

The population of neonates with swallowing and breastfeeding difficulties includes diverse subgroups requiring effective intervention strategies. Among the groups, oral feeding difficulties are prevalent in very-preterm infants at term-equivalent age (Grabill et al., 2023), subtle breastfeeding difficulties are found in late-preterm infants (Pike et al., 2017), and the majority of term neonates with hypoxic-ischaemic encephalopathy present with symptoms of oropharyngeal dysphagia after therapeutic hypothermia (Krüger et al., 2019). Oral feeding challenges are associated with neonatal co-morbidities and necessary clinical interventions while contributing to poor weight gain and delayed hospital discharge (Viswanathan & Jadcherla, 2020). Feeding difficulties may persist into childhood, cause anxiety in mothers (Walton et al., 2022) and predict poorer neurodevelopmental outcomes (Rinat et al., 2022).

Breastfeeding difficulties of preterm neonates are consistently reported in studies while their mothers display lower levels of breastfeeding self-efficacy at discharge, resulting in low rates of ongoing breastfeeding (Arvedson et al., 2020; Wang et al., 2019). Although the need for swallowing and breastfeeding interventions is clear, effectiveness should be considered.

Systematic reviews and meta-analyses reveal certainty of evidence and methodological problems not always apparent in individual studies. In a recent Cochrane systematic review and meta-analysis of 28 randomised controlled trials and 1831 preterm participants, Greene et al. (2023) found low- to very low certainty evidence for oral stimulation by finger when compared to standard care. Meta-analysis showed methodological concerns about allocation concealment and masking of caregivers and outcome assessors (Greene et al., 2023). It appears that oral sensorimotor interventions that include additional components such as non-nutritive suckling (NNS) show improved evidence even though studies still reveal methodological biases. In a meta-analysis, Chen et al. (2021) found that a broader intervention could increase feeding efficiency and weight gain, with reduced transition time to full oral feeds and length of hospital stay in preterm neonates. Evidence-based neonatal interventions from different disciplines could also be used by speech-language therapists to enhance effectiveness of oral feeding intervention.

One such intervention gaining consistent evidence over the past 30 years is kangaroo mother care (KMC). The practice involves skin-to-skin care, exclusive breastfeeding, early discharge and follow-up care (Chan et al., 2016). Kangaroo mother care has numerous benefits over conventional neonatal care, which include reducing mortality, nosocomial infection and sepsis, hypothermia, severe illness, lower respiratory tract disease while increasing weight, length and head circumference gain, exclusive breastfeeding, sleep organisation and mother‑infant attachment (Conde-Agudelo & Díaz-Rossello, 2016; Ludington-Hoe et al., 2006). In the past, KMC was recommended for stable infants only, but recent research indicates benefits extend to unstable neonates as well. A multisite study showed that immediate KMC after birth in a mother-neonatal intensive care unit (mother-NICU) reduced the mortality rate of low-birthweight infants by 25% (World Health Organization [WHO] Immediate KMC Study Group, 2021). The long-term effects of KMC for small neonates indicated significant differences in cognition, language and adaptive behaviour at 12 months corrected age (Bisanalli et al., 2023). Twenty years after KMC, young adults’ social performance and their parents’ protective behaviours showed significant differences over controls (Charpak et al., 2017). Kangaroo mother care is cost-saving to households and health systems and can be implemented in community settings (Choudhary et al., 2022). Since 2012, the South African Department of Health has endorsed KMC as an essential component of newborn care (Republic of South Africa, Department of Health, 2012). The WHO (2022, 2023) now views KMC as a transformative innovation in healthcare and recommends it as preventative and promotive care for preterm and low-birthweight infants immediately after birth.

Kangaroo mother care is clearly a valuable intervention, preparing mothers and infants for swallowing and breastfeeding intervention. Because mothers and infants are bonding and spending much time together, mothers get to know their infants well (Cho et al., 2016). Mothers may learn to read their signals intuitively. Experience in a KMC ward has shown that mothers are often very motivated to use interventions to prepare for safe oral feeding. A KMC ward, where continuous skin-to-skin care is practised, creates a social environment where lodging mothers can learn from health workers and one another and provide mutual support. Apart from rare exceptions, most mothers are available during feeding times. Alternative caregivers, such as grandmothers, may provide skin-to-skin care too. Mothers provide expressed breastmilk or may use donor breastmilk or preterm formula.

With exclusive breastfeeding as the goal, the latest breastfeeding and breastmilk science is already incorporated in KMC. Bottles and artificial nipples are avoided, protecting against complications such as oxygen desaturation and thrush (Stoltz & Bavousett, 2024; Thoyre & Carlson, 2003). Kangaroo mother care, as a single intervention, promotes lactation in mothers, reduces transition time from tube- to full oral feeding (Şimşek et al., 2023), improves neurodevelopment and has neuroprotective effects (Charpak et al., 2022). It is not known whether skin-to-skin holding on a mother’s bare chest has direct effects on infant oral sensorimotor abilities and the maturation of suck-swallow-breathing coordination. Further research is required.

In this article, we present a comprehensive neonatal swallowing and breastfeeding intervention programme based on existing evidence and clinical perspectives. The programme is intended for small neonates (preterm and low-birthweight), with modifications for sick term neonates.

We developed the programme while working as a speech-language therapist and medical doctor in an established KMC ward at a tertiary hospital. We hope to highlight the benefits of collaboration between mothers and the neonatal team during swallowing and breastfeeding intervention. We invite researchers to study the effect of the programme.

## Swallowing and breastfeeding intervention programme

**FIGURE 1:**
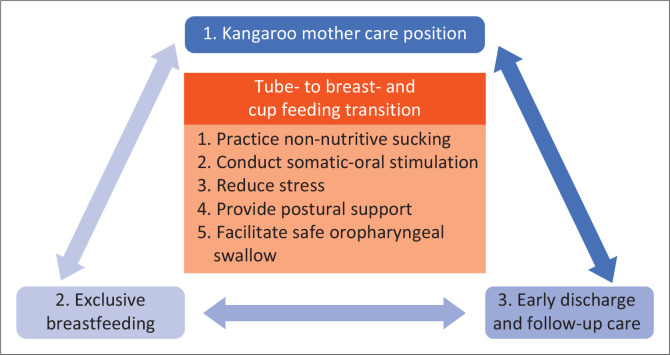
Swallowing and breastfeeding intervention framework for preterm, low birthweight and sick term neonates in kangaroo mother care.

The programme with its five sequential components is fully integrated in KMC as illustrated in Figure 1. We recommend practising intermittent KMC, but where possible, immediate KMC in a mother-NICU or continuous KMC in a KMC ward, while mothers follow the programme to guide their babies from tube- to breastfeeding and cup feeding. The implementation of the programme is dependent on assessment of breastfeeding readiness and tracking progress in the mother and infant, using a tool such as the Preterm Infant Breastfeeding Behavior Scale (PIBBS) (Nyqvist et al., 1996), and infant instrumental assessments when indicated.

Adjustments to the programme are made according to gestational age, birth- and current weight, chronological age (postnatal age), medical and feeding history and specific diagnoses like neonatal encephalopathy (NNE), genetic syndromes or other congenital conditions. All factors contributing to oral feeding difficulty, including those of mothers, should be considered. Mothers of very-preterm infants may experience the long period of transitioning to breastfeeding challenging and exhausting (Madiba & Sengane, 2021).

Feeding, whether by tube, breast or cup, is in the mother’s hands and arms and should remain so from the start of intervention. Mothers are coached individually or in groups. They are regularly monitored by a speech-language therapist or other healthcare team members who demonstrate the movements, almost without touching the infant. Coaching is an interactive activity where a mother is guided to understand her infant’s cues and adjust stimulation and calming techniques when indicated. Her opinions and intuitive knowledge of the infant are valued. She should be fully supported by the neonatal team. As neonatal feeding difficulties tend to persist, ongoing problems can be addressed during KMC follow-up care in a multidisciplinary outpatient clinic.

**FIGURE 2:**
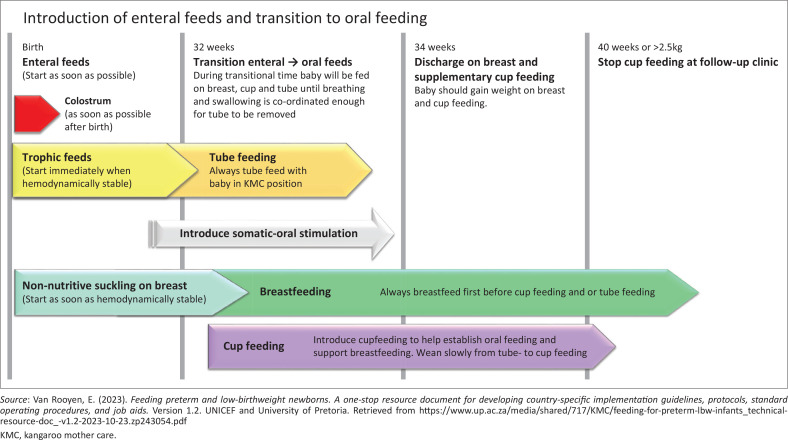
Timeline of when enteral feeds ideally should be introduced and the transition process from tube- to breastfeeding and cup feeding.

Figure 2 shows that early introduction of breastfeeding is key to the intervention programme. The timeline of introducing enteral feeds and transitioning to oral feeding is demonstrated by arrows in the diagram. At birth, enteral feeds should start as soon as possible. The red arrow displays the importance of giving colostrum to all small infants as soon as possible after birth. Enteral feeds are given in small volumes, also called trophic feeds. In very-preterm infants, all enteral feeds are given via gastric tube while infants are held in skin-to-skin position (Van Rooyen, 2023).

Non-nutritive suckling should be introduced as soon as the infant is haemodynamically stable. When the infant is more mature and shows signs of feeding readiness, breastfeeding is introduced. As soon as the infant is suckling on the breast, cup feeding is introduced. Tube feed volumes are slowly reduced and cup feed volumes are increased. The gastric tube is removed when the infant is gaining weight on both breast and cup feeds (Van Rooyen, 2023).

## Components of the programme

### Non-nutritive suckling

The first intervention component is eliciting the NNS reflex, an easier skill than the complex suck-swallow-breathing pattern of oral feeding. As infants swallow their own secretions only, NNS indirectly contributes to oral feeding, reducing demands on swallowing and energy expenditure (Barlow, 2009a). Non-nutritive suckling, although not predictive of oral feeding, is a precursor to nutritive suckling (Barlow, 2009b). Factors influencing NNS include degree of preterm birth, and medical complications such as patent ductus arteriosus and whether an infant was ventilated (Pineda et al., 2019). For these reasons, we recommend that the pathway to independent breastfeeding would start with NNS.

The most recent systematic review suggests that NNS, as a single intervention, shows significant benefits although the quality of study methods limited the credibility of findings (Foster et al., 2017). Subsequent studies confirmed that NNS reduces transition time from tube- to full oral feeding and from the start of oral feeding to full oral feeding, reduces length of hospital stay and increases physiologic stability but showed no positive effect on weight gain (Da Rosa Pereira et al., 2020; Pineda et al., 2019). It follows logic to prepare a neonate for oral feeding with NNS experience.

Non-nutritive suckling is safe for preterm infants under 30 weeks gestational age (Foster et al., 2017). As it represents the continuation of the foetal suck-swallow pattern, we recommend starting NNS soon after birth. The mother is coached to allow the infant to suckle on her empty breast or her clean little finger. The fingernail should be short with no sharp edges. Gloves are avoided as the smell, taste and feel may elicit aversive reactions in infants. The mother’s finger is safe, as mothers and infants share organisms during skin-to-skin contact.

When suckling on the empty breast, the infant is held in the breastfeeding position. Suckling on the mother’s little finger can be practised in the breastfeeding position or KMC position during tube feeding. Consistency in the infant holding pattern enhances learning experiences for both mother and infant. It appears that more infants suckling on an emptied breast achieve exclusive breastfeeding at discharge than infants suckling on pacifiers (Fucile et al., 2021). Non-nutritive suckling is a low-cost and safe intervention as no pacifiers are required. The recommended dosage is 3 min at a time, regularly during the day (Da Rosa Pereira et al., 2020).

### Somatic-oral stimulation

The aim of somatic-oral stimulation is to facilitate the complex suck-swallow-breathing pattern for safe and successful breastfeeding. The pattern originates in the brainstem by bilateral central pattern generators, functioning as endogenous interneuron circuits that require maturation in preterm infants (Barlow, 2009b). Central pattern generators are reinforced by feedback from sensory input on the skin (Arvedson et al., 2020). Barlow (2009b) indicates that oral sensorimotor stimulation prior to introducing oral feeds and NNS experience can activate central pattern generation and its muscle targets in the hyoid complex, accelerating independent oral feeding skills.

The somatic component of the stimulation sequence, involving stroking of the four limbs and trunk from the navel upwards in the direction of the mouth, serves to complement pre-feeding oral sensorimotor stimulation by finger (Online Appendix 1). The infant is removed from the KMC position and placed on a blanket in supine position on the mother’s bed. Subtle oral movements can usually be seen in the infant while conducting somatic stimulation. Fucile et al. (2011) found that very-preterm neonates who received stimulation of the limbs, trunk and perioral area achieved independent oral feeding significantly earlier than those with oral stimulation only. The hypothesis is that there may be distributed effects associated with somatic stimulation that go beyond the perioral area, as oral feeding involves multisystem functioning (Fucile et al., 2012).

Somatic stimulation by hand or three fingers is directly followed by a series of perioral and intraoral stroking by finger (Online Appendix 1). Oral stimulation facilitates suck-central pattern generators and activates rooting, the transverse tongue reflex, NNS and nutritive suckling (Arvedson et al., 2020; Barlow, 2009b). To avoid overstimulation, all stroking movements are carried out twice only, with gentle but firm pressure on the infant’s skin. The recommended dosage of somatic-oral stimulation is 3 min before each of the five daytime feeds, with a total of 15 min per day (Lessen Knoll et al., 2019).

During perioral and intraoral stimulation, the infant should be securely wrapped in a lightweight blanket to prevent hypothermia. When wrapped, the infant’s hands should be close to the face to promote hand-to-face exploration. Infants with NNE and those with hypersensitive responses should be stimulated with caution or avoid somatic-oral stimulation. Alternately, apply deep sustained pressure, starting on the periphery (trunk and limbs), while slowly moving towards the mouth, using the infant’s own hand or the mother’s (Arvedson et al., 2020). Try to activate NNS as it promotes stability, or proceed slowly with further stimulation.

After conducting the somatic-oral stimulation sequence, the very-preterm infant first suckles on the mother’s empty breast to gain NNS experience. The mother’s expressed or donor breastmilk is given via tube in the KMC position. In the more mature neonate, the infant is put on the breast for a breastfeeding session that may last for a few minutes only. If the infant is already transitioning to cup feeding, the specified volume is offered in the cup directly after the breastfeeding session. When the specified volume is not ingested by cup, the remainder of the milk is given by tube. Infants are wrapped and held in the cradle-hold breastfeeding position for cup feeding.

### Reduce stress in mothers and infants

The interaction of neonatal disease and clinical interventions causing pain and discomfort may decrease the infant’s ability to adapt to stressors (Casavant et al., 2019). Loud noise, bright light and unsupported infant handling may further contribute to stress. Loud ambient and sudden noise have immediate effects on the cardiovascular and respiratory systems of small neonates (Wachman & Lavav, 2010).

Infant stress cues are numerous and varied (Online Appendix 1). Mothers are coached to identify behaviours like irregular breathing, yawning, gagging, choking, hiccupping, staring, facial grimacing, squirming, arching and crying as stress cues, not as natural states. Noise and stress interfere with feeding, evidenced when an infant shows resistance to the presentation of the breast (Arvedson et al., 2020; Nyqvist et al., 1996).

Continuous KMC is effective to manage stress in infants and mothers as oxytocin, released during skin-to-skin touch, decreases cortisol secretion and reduces stress in both infant and mother (Pavlyshyn et al., 2022). When stress cues are observed during somatic-oral stimulation, NNS and the KMC position can be used to allow calming and stabilisation. Mothers should be coached to handle infants gently with slow movements during diaper changes and bathing. Infants should be turned sideways to be picked up and be supported with both hands. Containment for calming involves gently placing cupped hands around the infant (Arvedson et al., 2020).

Infants in NICUs are overexposed to the smells, taste, touch, visual images and noise of a clinical environment but deprived of natural sensory experiences. Kangaroo mother care offers an alternative. In the clinical NICU and KMC environment, staff and mothers are encouraged to reduce noise and bright lights and avoid visual distractions like bold colours and pictures on the wall. The closeness of the KMC position creates a consistent biological, sensory and social environment, enabling the first steps of learning. The infant becomes familiar with the mother’s distinctive smell, taste and smell of her breastmilk, her touch, rhythm of her movements, face and voice.

Instead of noise, infants should hear their mothers’ voices. It appears that the release of oxytocin is not only influenced by touch but also by vocal stimulation (Hirschel et al., 2023). Mothers are encouraged to talk, sing and read with their infants during KMC, as a combination of touch and vocal stimulation decreases cortisol and increases saliva oxytocin levels in mothers (Hirschel et al., 2023). Talking with her infant is comforting for both while enhancing bonding. Hearing the maternal voice is also necessary for the preterm neonate’s auditory system development and language learning (Graven & Browne, 2008).

### Provide postural support for mother and infant

As a result of decreased oral reflexes and hypo- or hypertonicity, small and sick neonates require continuous postural support during tube-, breastfeeding and cup feeding and general handling. We recommend just two infant holding positions for feeding: the KMC position for tube feeding and the cradle-hold position for both breastfeeding and cup feeding. Guidelines for infant holding during cup feeding are often lacking in scientific literature, which could have contributed to illustrations seen on marketing material where infants are supported by the neck only. A mother’s body and arms should be supported by a chair with armrests, so that she can support her infant appropriately for the full duration of a feeding session. She should be sitting comfortably with feet on the floor while supporting the infant in cradle-hold.

The mother supports the infant’s body with her abdomen and arm, while the infant’s head rests securely in the angle of the bent elbow of the other arm. This position allows infant head-body alignment, with arms and shoulders slightly forward, chin near the chest (but not too close to cause pharyngeal obstruction) and hips flexed. A flexed body posture facilitates body alignment and movement (Wahyuni et al., 2022). This study showed that physiological flexion postural tone was the most important factor influencing oral feeding ability in preterm infants.

### Facilitate safe oropharyngeal swallowing

Infant-led feeding is recommended. We observe less choking and coughing during breastfeeding than with cup feeding, as infants can better pace their milk intake. When milk flows too fast with cup feeding, prolonged holding of the bolus in the mouth, delayed swallowing and coughing are regularly observed, resulting in milk spillage and loss of volume ingestion. Mothers are guided to reduce the milk flow during cup feeding, similar to what Lau and Smith (2012) describe as minimal bolus swallow. Additionally, the mother periodically presses her finger on the soft tissue under the infant’s chin to stabilise the jaw and facilitate lip closure and swallowing.

With suck-swallow-breathe incoordination, preventing acute and chronic respiratory aspiration while ensuring safe swallowing is of primary importance. We avoid syringe feeding for two reasons: high risk of aspiration as it is difficult to regulate bolus size, and no suckling experience is gained. Using a 10- or 20-mL syringe for feeding may result in injecting a bolus too large to swallow and causing aspiration. Bottle feeding may also render large boluses. Hernandez and Mandelbaum Gonçalves Bianchini (2019) showed with videofluoroscopy swallow studies that bottle feeding is associated with laryngeal penetration, tracheal aspiration and gastrointestinal reflux in comparison with breastfeeding. Negative experiences with coughing and choking may cause aversive reactions to oral feeding attempts (Arvedson et al., 2020). Such reactions are observed when the phasic bite reflex is elicited instead of a suckling reflex while touching the infant’s gums (Arvedson et al., 2020).

## Conclusion

Feeding a small or sick infant is challenging; it takes much patience and the mother may find it exhausting with little progress perceived. The intervention programme places feeding into the mothers’ hands, and with support and coaching from the healthcare team they can learn to hold and breastfeed their small or sick infants while observing progress. The authors suggest that swallowing and breastfeeding intervention is enhanced by the practice of KMC.

The programme described in this paper is based on available evidence for its individual components that are not harmful to a neonate. Evaluating the effectiveness of a complex feeding intervention is challenging. Further studies could include measuring outcomes such as PIBBS scores, growth, duration of hospital stay, prevalence and duration of exclusive breastfeeding and healthcare workers’ and mothers’ experiences of the programme.
